# U.S. adults’ attitudes toward abortion as a (non)essential procedure during the COVID-19 pandemic

**DOI:** 10.1186/s12889-025-23266-2

**Published:** 2025-06-07

**Authors:** Kyla M. Cary, Kristen N. Jozkowski, Brandon L. Crawford, Ronna C. Turner, Danielle Layton, Chloe Lohrmann, Jacqueline Y. Paiz

**Affiliations:** 1 Department of Applied Health Science, School of Public Health-Bloomington, Indiana University, Bloomington, IN 47405 USA; 2 The Kinsey Institute for Research in Sex, Gender, & Reproduction, Indiana University, Bloomington, USA; 3 Educational Statistics and Research Methods, College of Education and Health Profession, University of Arkansas, Fayetteville, USA

**Keywords:** Abortion attitudes, Essential procedure, COVID-19, Abortion healthcare

## Abstract

**Background:**

The American College of Obstetricians and Gynecologists (ACOG) state “abortion is healthcare” in their advocacy statement. During the COVID-19 pandemic, healthcare services were limited to those deemed “essential,” and some conservative states’ attorneys general classified abortion as “nonessential,” against ACOG recommendations. Subsequent to the pandemic, the 2022 U.S. *Dobbs v. Jackson Women’s Health Organization* decision eliminated the federal right to abortion, and many states have severely restricted abortion access, making it important to ask—do people consider abortion essential healthcare? Thus, we explored U.S. adults’ opinions on abortion as an essential medical procedure.

**Methods:**

We collected data via IPSOS’s KnowledgePanel^®^ from September 2020 to January 2021 in four waves, three of which are included in the present study. We asked, “Do you think abortion is an essential medical procedure?” (yes, no, unsure) with a follow-up open-ended question: “Please describe why you [think/do not think/are unsure] abortion is an essential medical procedure.” Our sample comprised 1523 closed-ended and corresponding open-ended responses. We used descriptive statistics to examine closed-ended responses by wave and inductive qualitative content analysis to analyze open-ended responses collapsed across waves.

**Results:**

Among closed-ended responses, 44–46% indicated abortion was an essential procedure, 32–35% indicated abortion was not essential, and 19–22% indicated unsure. Among open-ended responses, 49% stated abortion was an essential medical procedure because abortion is time-sensitive, a right, and prevents suffering. Of open-ended responses, 43% indicated the essentiality of abortion as a medical procedure depends on the reason. And 32% of open-ended responses said abortion is not an essential medical procedure because abortion is elective, wrong/murder, and other procedures should take precedence. We noted instances where closed-ended responses did not correspond with open-ended reasons.

**Conclusions:**

Findings highlight the nuance in conceptualizing abortion as an essential medical procedure. Reasons provided by participants on the extent abortion is an essential medical procedure underscore the contextual nature of abortion attitudes found in previous research. Notably, people indicating abortion was not an essential medical procedure but providing explanations suggesting reasons to consider abortion essential, point to the need for more nuance in assessing abortion attitudes.

## Background

In an advocacy statement, the American College of Obstetricians and Gynecologists (ACOG) states that “abortion is healthcare” [1], and some public opinion polls include items that frame abortion as healthcare (e.g., How important will each of the following ***healthcare issues*** be in deciding your vote for president this year? [emphasis added]; Will determining the future of reproductive ***health issues including abortion*** be very important, somewhat important, not too important, or not at all important? [emphasis added]) [2]. But, to what extent does the United States (U.S.) public conceptualize abortion as healthcare?

During the COVID-19 pandemic, limitations were placed on healthcare services as a means for conserving resources, such as hospital beds and personal protective equipment (PPE). Specifically, healthcare services were limited to only those considered “essential” [3], with classifications of essential procedures varying from state-to-state [4]. Indeed, in developing state responses to the need to triage healthcare services (because of COVID-19), some conservative states’ attorneys general classified abortion as nonessential [5]. Abortion provider organizations have argued against such restrictions for myriad reasons, notably that abortion is a necessary aspect of healthcare [6].

Importantly, these COVID-related restrictions occurred prior to the overturning of *Roe v. Wade*; the 1973 U.S Supreme Court decision that established a constitutional right to abortion and prevented states from banning abortion prior to fetal viability. However, the Court’s 2022 decision in *Dobbs v. Jackson Women’s Health Organization* (i.e., *Dobbs*) overturned that right, resulting in shifts in the abortion legislative landscape such that states can and have fully or partially restricted abortion earlier in pregnancy than previously permitted [7]. These legal changes may have fueled further awareness and public discourse of the state of abortion access in the U.S [8, 9]; however, research assessing people’s attitudes toward abortion in the context of healthcare is limited. As such, in the present study, we sought to examine the U.S. public’s opinions on whether abortion should be considered an essential medical procedure, which provides insight regarding people’s perceptions of abortion as healthcare. The study was conducted prior to Dobbs but is still highly relevant today.

### Guidelines for essential procedures during COVID-19

The emergence of COVID-19 as a global pandemic in late 2019 greatly shifted the global healthcare landscape. As COVID-19 incidences continued to rise, on March 11, 2020, the World Health Organization (WHO) declared COVID-19 to be a global pandemic [10]. In the U.S., in anticipation of depleting resources, medical organizations released recommendations for triaging care and conserving medical resources, including identifying top-priority, or “essential,” medical procedures [3, 11, 12]. For example, the federal Centers for Medicare and Medicaid Services (CMS) provided recommendations on a three-tier scale. Tier 3 included high acuity treatment or services for which lack of in-person treatment or service would result in patient harm; the CMS did not recommend postponing such in-person evaluation. Tier 2 included intermediate acuity treatment or services in which not providing the service had the potential for increasing morbidity or mortality, recommending initial evaluation via telehealth, with triage to appropriate sites of care. Tier 1 included low acuity treatment or service, care for which could be postponed [3]. The American Medical Association (AMA) and WHO also released similar guidelines [11, 12].

Additionally, on March 17, 2020, the U.S. American College of Surgeons (ACOS) recommended the cancellation of all “elective” surgeries [13]. However, as Prachand and colleagues [14] described, the term “elective” included many medically necessary, time sensitive procedures, which contributed to confusion and decision difficulty among both medical professionals and the general population. Procedures classified as “elective” included those such as cancer surgeries and treatments of peripheral vascular disease, where approval was based on factors such as disease severity, time sensitivity, and the availability and projected outcome of nonoperative alternatives [14]. Thus, the pandemic challenged what was and was not classified as essential including, but not limited to, abortion, and individual states began developing policies in response to guidelines put forth by medical associations.

### Abortion as a (non)essential service

Although CMS, AMA, and WHO provided recommendations for determining essentiality, identifying which individual services fell into the category of essential was largely up to individual states [15], and some states’ attorneys general were quick to make a distinction for abortion services. As one example, a nationwide lockdown went into effect in the U.S. on March 15, 2020 [10], and in the earliest days of the lockdown, on March 21, 2020, Ohio’s Republican attorney general ordered clinics to halt abortion services with a statewide measure to conserve healthcare resources [16] despite there being only 147 confirmed COVID cases in Ohio at the time [17]. Around the same time, Texas’s Governor issued executive order GA9 to postpone all surgeries and procedures that were not “immediately medically necessary,” which did not originally reference abortion; however, the attorney general confirmed that abortion was deemed “nonessential” unless to preserve the life of the mother [5]. In the next several weeks, several states followed Texas and Ohio’s lead, declaring abortion as nonessential and restricting access as part of emergency orders against elective or nonessential medical procedures, including Mississippi, Iowa, Indiana, Oklahoma, and Alabama [18, 19].

Actions taken by states to limit abortion access, arguing it was not essential, conflicted with the recommendations of several medical associations. For example in a joint statement from eight medical groups, including ACOG and the American Board of Obstetrics and Gynecology, medical providers argued that abortion is a time-sensitive service, as delaying access for any amount of time may increase risk or make the service inaccessible [20]. Additionally, in their published interim guidance titled, “Maintaining essential health services,” the WHO specifically addressed sexual and reproductive healthcare as essential healthcare, highlighting the potential maternal and newborn deaths that would occur due to unintended pregnancies, unsafe abortions, and high-risk deliveries resulting from limited sexual and reproductive health services during the pandemic. As such, the WHO recommended reducing barriers that would delay abortion and promoting the provision of noninvasive methods for safe abortion, including telemedicine and delivery of medication abortion [12]. However, this interim guidance was not officially published until June of 2020, by which time abortion care restrictions were already underway in several states.

Advocates and organizations supportive of and opposed to abortion access also voiced opinions on COVID-related abortion restrictions. For example, anti-abortion advocates argued that ceasing abortion services was necessary to divert medical resources toward responding to the COVID-19 pandemic [17]. In contrast, the WHO recommendations for telemedicine and delivery of medication abortion [12] would arguably reduce the need for hospital beds and resources required for prenatal and pregnancy-related healthcare needs, including birth. In addition, abortion providers and advocates questioned the medical necessity of limiting abortion access, arguing that COVID-related efforts to restrict abortion services were part of a political agenda to restrict and/or ban abortion altogether [16]. Notably, many of the states that were quick to define abortion as a nonessential procedure already had more restrictive abortion laws relative to many other states [21]. Indeed, in April 2020, Planned Parenthood president Alexis McGill voiced that the pandemic was “being used as a cover” to restrict abortion access [6].

### U.S. adults’ attitudes toward abortion as healthcare

Abortion advocates identified states’ COVID-19-related abortion restrictions, based on the assertion that abortion is not essential healthcare, were part of an effort to remove abortion rights on a national scale [6, 22, 23]. Most medical professionals argue that abortion is a key aspect of reproductive healthcare and should therefore be considered essential healthcare [24]. However, the perception of “abortion as healthcare” among the U.S. public remains largely unexplored.

Most commonly, public opinion on abortion is assessed through national poll or survey items that ask about the legality of abortion, whether it should be legal in all cases/circumstances, legal/illegal in some cases/circumstances, or illegal in all cases/circumstances. For review of polling items, see Bowman and Goldstein [25]. Framing abortion mostly as a legal issue may not fully capture the public’s perspective on abortion [26, 27]. Further, exploring the public’s conceptualizations of abortion as healthcare has significant public health implications, as evidenced in the context of the COVID-19 pandemic and the reduction of healthcare services to only those considered “essential.” Therefore, in this study, we examined a unique aspect of people’s attitudes toward abortion in terms of conceptualizing abortion as an essential medical procedure. Findings can provide insight as we move forward in a post-*Dobbs* context where abortion access is limited in many U.S. states.

### The present study

The aim of the present study was to assess U.S. adults’ attitudes toward whether abortion should be considered an essential medical procedure during the COVID-19 pandemic and their rationale for holding these attitudes. With this aim, we gleaned ancillary information about people’s perceptions of abortion as healthcare. We explored the following research questions:

RQ1: Do U.S. adults consider abortion an essential medical procedure?

RQ2: What reasons do U.S. adults offer for why abortion should or should not be considered an essential medical procedure?

## Methods

### Study design and data collection

In 2020, we launched an online survey using IPSOS’s probability-based KnowledgePanel^®^, a web-based panel designed to be representative of the U.S. household population. The purpose of the overarching study was to examine if and how attitudes toward abortion shift during an election cycle, particularly if, and when, abortion comes up in a meaningful way during the election year. All data collection procedures were approved by the Institutional Review Board at Indiana University, and informed consent was obtained from all participants before participating. Data collection occurred simultaneously in English and Spanish, with linguistically and culturally congruent survey versions developed for both languages using a co-construction model with English as the source language [28]. Data collection occurred in four waves: Wave 1 (*N* = 919) was collected in September 2020, Wave 2 (*N* = 711) in October 2020, Wave 3 (*N* = 637) in December 2020, and Wave 4 (*N* = 576) in January 2021. Items relevant to this study were included in Waves 2, 3, and 4. Participants’ responses across waves were collapsed for analysis (see description in our Analytical Sample), thus the present analysis treats data as cross-sectional.

The online survey comprised approximately 130 items, including items assessing demographic information, attitudes toward and knowledge of abortion laws, and attitudes toward the legality and morality of abortion. Relevant to the present study, two survey items pertained to attitudes toward whether abortion is an essential medical procedure—one closed-ended and one open-ended; these items were provided toward the end of the survey. The closed-ended survey item asked, “*During the coronavirus outbreak*,* medical appointments were limited to****only those considered essential***. *Do you think abortion is an essential medical procedure?*” with response options of yes, no, and unsure. Depending on a participant’s response to the closed-ended question, participants were asked a follow-up open-ended question, “*Please describe why you [think/do not think/are unsure] abortion is an essential medical procedure.”* Participants typed their open-ended responses into a text box following the question. All survey items relevant to this study are provided in the Appendix. On average, participants took approximately 20 min to complete the full survey.

### Analytical sample

Our analytical sample comprised all responses entered by participants for the above closed-ended and open-ended items collected during Waves 2–4 of data collection. In our sample, 643^1^ participants provided a response to the open-ended textbox in at least one of the study waves, with many responding to the question at multiple waves, totaling 1523 responses across the three waves. Open-ended responses were typically brief (i.e., one to two sentences in length); the mean number of words per response was 14.65 (*SD* = 14.59). Of these responses, 575 were collected at Wave 2 (557 English, 18 Spanish), 493 responses were collected at Wave 3 (478 English, 15 Spanish), and 455 responses were collected at Wave 4 (442 English, 13 Spanish). Participants in our sample (*N* = 643) ranged in age between 18 and 94 years when data were collected in Wave 2 (*M* = 52.93, *SD* = 17.36); 333 (51.8%) identified as men and 310 (48.2%) identified as women. Additional participant sociodemographic information is provided in Table 1.

**Table 1:** Participant sociodemographic information (*N* = 643)

Variable	*N* (%)
**Race/ethnicity**	
Non-Hispanic White	496 (77.1)
Hispanic White	39 (6.1)
Non-Hispanic Black/African American	54 (8.4)
Hispanic Black/African American	2 (0.3)
Non-Hispanic Other	49 (7.6)
Hispanic Other	3 (0.5)
**Education**	
Less than high school	42 (6.5)
High school	170 (26.4)
Some college	178 (27.7)
Bachelor’s degree or more	253 (39.3)
**Political affiliation**	
Republican	201 (31.3)
Democrat	207 (32.2)
Independent/other	235 (36.5)
**Abortion Identity**	
Pro-life	239 (37.2)
Unsure	68 (10.6)
Pro-choice	336 (52.3)
**Religious attendance**	
More than once a week	51 (7.9)
Once a week	125 (19.4)
Once or twice a month	33 (5.1)
A few times a year	78 (12.1)
Once a year or more	120 (18.7)
Never	172 (26.7)
Did not respond	64 (10.0)
**Biblical literalism**	
It is the word of God, to be taken literally	162 (25.2)
It is the word of God, but not everything should be taken literally	239 (37.2)
It is a book of history, not the word of God	38 (5.9)
It is a book of stories, written by people	151 (23.5)
I do not know	53 (8.2)

*Note.* Age, gender, race/ethnicity, and education information provided by IPSOS; religion items derived from initial Wave 1 data collection; political party affiliation and abortion identity information derived from Wave 2 data collection

In the present study, we combined and analyzed responses provided across all waves, thus the unit of analysis within the present study is participant responses to both the closed-ended and open-ended questions. We note that this includes multiple responses from a single participant provided across multiple waves. We elected to combine all responses across waves in our analysis for the following reasons [1] nearly half of participants (*N* = 292) did not complete all three waves [2], when they did complete a wave, not all participants chose to provide an open-ended response, and most importantly [3], participants were able to provide different responses to the closed-ended item across waves, and thus may have responded to a different follow-up open-ended item in each wave, as the open-ended items were programmed to correspond with closed-ended responses. Indeed, approximately 20% of participants who responded to more than one wave provided different responses to the closed-ended item across waves. Among these participants, we found variation in observed patterns of responding. Some participants shifted toward “unsure” across waves, others shifted away from “unsure” in earlier waves to “yes” or “no” in later waves, and some participants provided a different response option at each timepoint. The inclusion of responses from multiple waves of data provided the ability to capture both initial thoughts and responses potentially informed by contemplation due to repeated prompts from multiple survey administrations. We note that our aim in the present study was not to explore changes in attitudes toward abortion as essential. Rather, we sought to descriptively examine reasons provided for *why* abortion should or should not be considered an essential medical procedure. Given that our goal was to elicit participants’ rationale for considering abortion essential/nonessential, we can potentially achieve a broader perspective on this issue by including multiple waves of data.

### Analytical approach

In addition to analyzing descriptive statistics corresponding to the closed-ended item, we conducted a inductive qualitative content analysis of open-ended responses [29]. The coding team consisted of four researchers [KC, DL, CL, JP] who engaged in coding and analysis; three researchers are native English speakers [KC, DL, CL], and one researcher is a native Spanish speaker and bilingual in English [JP]. Data were not translated; all data were analyzed in the original language used by respondents. Our analytical process followed an inductive approach defined by three phases (1) preparation, (2) organizing, and (3) reporting [29]. During the preparation phase, we determined the unit of analysis to be open-ended responses provided across the three waves of data collection, as is discussed above. The organization phase began with an open coding process to make notes and develop initial categories of responses, followed by solidifying and collapsing categories, and ending with abstraction or formulating a general description of our research topic through naming and interpreting categories. In the final reporting phase, we prepared a report of our analytical process and results.

We began open coding by individually examining a subsample of the 1523 responses (i.e., 30%, *n* = 457). This subsample of responses was split evenly between two pairs of coders [KC & DL, CL & JP], and each pair of coders examined approximately one-half of subsample responses). Due to the small number of Spanish responses, the bilingual team member [JP] also analyzed 100% (*n* = 46) of Spanish responses. Each coder engaged in independent reading of responses and memoing to generate emerging categories of responses which resulted in creation of individual preliminary codebooks. During this exploratory phase, the full four-member team analyzed responses separately based on participants’ response (yes, no, unsure) to the closed-ended item (“*Do you think abortion is an essential medical procedure?)* to assess whether separate codebooks should be generated and used for each group of responses. We sought to identify emerging patterns of responses as well as assess potential differences in such patterns between those who responded yes, no, and unsure to the closed-ended item. We determined that there were significant overlaps between open-ended responses, regardless of participants’ closed-ended item response, and thus determined it appropriate to develop a single codebook to be applied to all responses.

After each coder developed their initial independent codebook, the first author [KC] reconciled individual codebooks and memoing into a harmonized initial team codebook and generated suggested operational definitions for codes and larger themes. The full coding team then met to collaboratively discuss the harmonized codebook and made adjustments during intensive review and discussion. This process led to the development of an initial collective codebook that was then used to engage in another round of coding of a second subset of responses. All coders coded a new subsample of 30% (*n* = 457) of all English responses, while the Spanish-speaking coder coded a subsample of 50% (*n* = 23) of Spanish responses. The 30% of English responses were split evenly among two pairs of coders, allowing for application of interrater reliability. We used STATA to calculate Cohen’s kappa estimates to assess agreement between pairs of coders for each individual code, with lower kappa coefficients indicating poor agreement between coder pairs. The full coding team then met again to review and reconcile discrepancies in coding between coders, particularly for codes with low kappa estimates, and come to consensus on code definitions. This process continued until a collective understanding of codes and definitions was achieved and a final codebook was generated (see Table 2).

**Table 2:** Themes, codes, and definitions

Theme	Code	Definition
	Uncodeable	Unintelligible response, no response, no reason provided, errors/typos, doesn’t answer the question of essentiality
Abortion is Essential	Abortion is essential	General statements that abortion is essential
	Abortion is time sensitive	Abortion is essential because it is time sensitive / waiting to have an abortion can lead to the procedure becoming illegal / abortion should be sought early
	Women’s right to choose / women’s choice	Abortion is essential because women have the right to choose / should have bodily autonomy
	Essential as women’s healthcare	Abortion is essential as an aspect of women’s reproductive healthcare
	Essential to prevent suffering	Abortion is essential to prevent negative outcomes for the woman and/or child
Abortion is Not Essential	Abortion is not essential/necessary	General statements that abortion is not necessary / medically necessary
	Abortion is an elective procedure	Abortion is not essential because it is an elective procedure
	Abortion is wrong	Abortion is not essential because abortion is morally wrong
	Abortion is murder	Abortion is not essential because it is murder
	Abortion should not be used to avoid responsibility	Abortion is not essential because it is used as a means to avoid responsibilities of parenting / abortion should not be used as birth control / pregnancy can be prevented
	Alternatives to abortion	Abortion is not essential because, once born, the child can be adopted
	Other medical issues are more important	Abortion is not essential because there are other medical procedures that should take precedence
	Abortion is not time sensitive / can be delayed	Abortion is not essential because it is not time sensitive / can be delayed
Essentiality Depends	Essentiality depends on the reason	Essentiality of abortion depends on circumstances/situation/reasons; abortion should only be done for valid reasons (applied to responses that did not specifically state a reason such as health, rape)
	Essential in cases of rape/incest	Abortion is only essential in cases of rape
	Essential if medically necessary	Abortion is only essential when it is done to protect the health or life of the mother / due to fetal anomaly
Don’t Know, Unsure, or Unable to Decide	Don’t know unsure, or unable to decide	Participant doesn’t know enough information to comment / is unsure of their own attitudes towards abortion / is unable to make a decision

The coding team then applied the finalized codebook to all 1523 responses. The sample was coded with 30% overlap, such that 30% (*n* = 443) of all English responses were coded by all four coders. Remaining responses were distributed evenly among each coder to be independently coded. Interrater reliability statistics were calculated among responses coded by multiple coders (i.e., 30% of English responses). Results reflected an acceptable degree of agreement with Cohen’s kappa estimates for codes averaging between 0.69 and 0.75 among each possible pair of coders.

## Results

Below, we present closed-ended and then open-ended findings. Of note, participants’ responses to the open-ended question did not always align—completely or partially—with their closed-ended response. The ways in which partial or complete misalignment manifested were sometimes nuanced. Thus, we follow with a comparison of closed- and open-ended responses.

### Findings from closed-ended responses

Frequencies of closed-ended responses for each wave along with weighted percentages^2^ are provided in Table 3. Among closed-ended responses and across waves, 44–46% indicated that abortion is an essential medical procedure (i.e., responded “yes” to the closed-ended item), 32–35% indicated abortion is not an essential medical procedure (i.e., responded “no” to the closed-ended item), and 19–22% indicated unsure about whether abortion is an essential medical procedure (i.e., responded “unsure” to the closed-ended item).

**Table 3:** Frequencies of closed-ended responses by wave

Closed-ended Response	Wave 2 *N* (weighted %)	Wave 3 *N* (weighted %)	Wave 4 *N* (weighted %)
Yes	267 (45.5)	225 (45.6)	205 (44.0)
No	185 (35.2)	149 (32.0)	149 (34.7)
Unsure	123 (19.3)	119 (22.4)	101 (21.3)

### Findings from open-ended responses

**Fig. 1:**
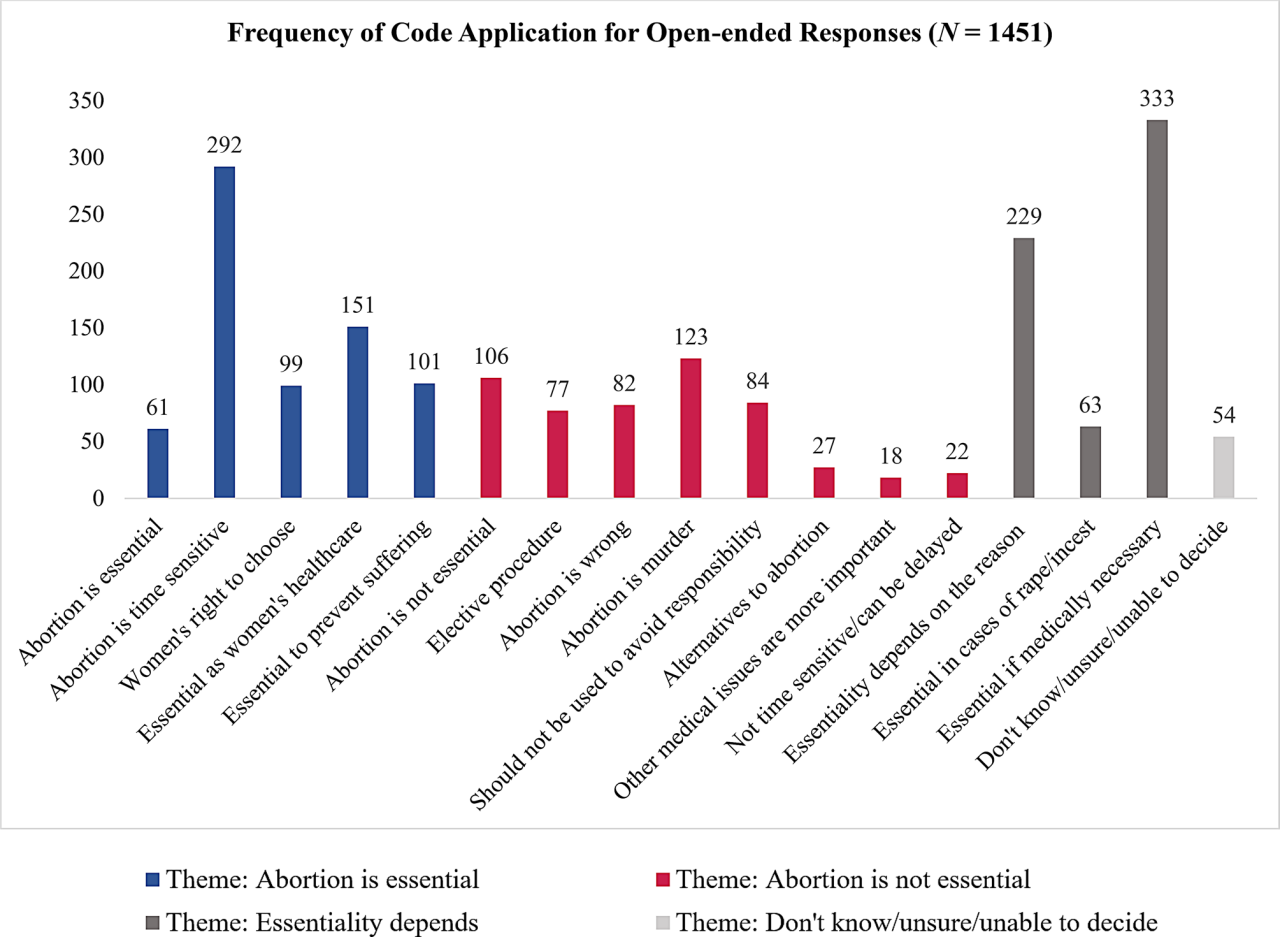
Frequency of individual code application across open-ended responses (*N* = 1451) to the item: “Please describe why you [think/do not think/are unsure] abortion is an essential medical procedure”

Of the 1523 total open-ended responses, 72 could not be coded (see Uncodeable code definition in Table 2), thus leaving a sample of 1451 content responses that were coded. More than one code could be applied to each response, thus reported frequencies can total greater than 1451, and reported percentages can total greater than 100. See Fig. 1 for frequencies of application for each individual code. Below we outline prevalence of themes and individual codes, describe participant responses, and provide unaltered exemplar quotes. We also compare whether reasons provided in response to the open-ended item conflicted with closed-ended responses.

### Qualitative themes

Responses to the open-ended question reflected a similar pattern to the options provided in the essential medical procedure closed-ended question (i.e., yes, no, unsure) with identified themes of ‘Abortion is essential,’ ‘Abortion is not essential,’ ‘Essentiality depends,’ and ‘Don’t know, unsure, or unable to decide.’ Although in most instances, codes applied to open-ended responses aligned with closed-ended responses, notably, some responses did not align across the two sets of questions (e.g., response to the close-ended item indicated that abortion is not an essential medical procedure, but the open-ended response indicated that the extent that participants consider abortion an essential medical procedure may depend). Each theme and corresponding individual codes are discussed below with accompanying verbatim exemplar quotes, followed by examination of conflicting responses.

### Abortion is essential

The theme ‘Abortion is essential’ was applied the most frequently (48.5% of responses within full sample) and encompassed reasons why abortion should be considered an essential medical procedure. Responses underlying this theme constructed essentiality primarily by drawing upon rationales of urgency, healthcare, and ethics. While only one of these rationales foregrounds abortion as an essential medical procedure, responses suggest that legal and ethical reasons for essentiality contribute to positioning abortion as healthcare as well.

This theme’s most prevalent individual code (applied to 20.1% of responses within full sample) comprises responses which framed abortion as an essential medical procedure because it is *time sensitive*. Such responses reflect discussion of urgency: people seeking abortion should be able to access it quickly for reasons of legality and health, among others. Responses stated that abortion should be accessed “sooner than later” and “if you need an abortion, you need to have it done in a timely manner.” In addition to these more general statements, participants also referenced time sensitivity in relation to legislation that places time limits on the legality of abortion: “Time, given the current law, is an essential factor in whether or not the procedure is legal.” Time sensitivity constructs medicality^3^ [30] and legality as mutually constitutive because navigating legal restrictions on abortion necessitates framing abortion as a medical necessity and/or prioritizing earlier care. Participants also stated that waiting to access abortion could result in unwanted birth and potential medical complications, “waiting too long could require the woman to end of having to give birth. Also waiting could cause the woman more complications than having the procedure when she first wants to get it done.” Similarly, participants noted that, given a situation in which the pregnant person’s life is in danger, abortion would need to be accessed immediately.

The next two codes, though notably not the most prevalent, construct abortion as an essential medical procedure by explicitly constructing abortion as a part of healthcare. The code *essential as women’s healthcare* (applied to 10.4% of responses within full sample) comprised statements such as, “It is a reproductive health care issue like any other health concern,” and “Women’s reproductive health is essential healthcare.” Similarly, the individual code *abortion is essential* (applied to 4.2% of responses within full sample) captures general statements that abortion is an essential medical procedure: “All medical services are essential,” “Because it is,” “Need for abortion doesn’t change because of the pandemic.”

Remaining codes comprise responses that constructed abortion as an essential medical procedure through rationales related to ethics: abortion may be sought to prevent suffering and curbing access to abortion violates autonomy. Responses reasoned that ending an unwanted pregnancy *prevents suffering* (applied to 7% of responses within full sample). Framing abortion as essential to prevent suffering constructs an entangled relationship between the medicality and ethicality of abortion as it included preventing suffering both for an unwanted child: “If a woman can not [sic] accept her pregnancy for any reason how can anyone expect her to give loving care to her child (for ever)?” as well as for the would-be parent, “Some mothers may need it to save their life. Some may have lost their jobs and can’t afford a baby due to the pandemic.” Relatedly, other responses spoke to the seriousness or gravity of abortion decision-making to rationalize essentiality. For example, some responses noted that the decision to seek an abortion is “life changing” and thus necessitates essentiality, “Because it can have a serious effect on others lives,” and “Because it will effect the life of the parents forever and so is permanent and essential.” Responses that placed weight on the significance of abortion decision-making suggest that abortion may impact people’s lives, which could be implicitly related to health and wellbeing, but these responses did not directly link abortion to healthcare.

Constructing essentiality based on ethics also focused on preserving human rights. Respondents framed abortion as an essential medical procedure because not considering it so infringes upon *women’s rights and bodily autonomy* (applied to 6.8% of responses within full sample): “Her body, her choice,” “Critical right that women have complete control over their bodies,” and “If a woman needs it [abortion], she should be able to have the procedure and it should be her choice not some old Man [in] Washington and a pervert president who lies about everything that has been married three times.” Foregrounding the ethics of preserving rights and bodily autonomy implicitly frames abortion as healthcare while taking the moral stance that whether the abortion is “indicated” or “elective” is irrelevant.

### Essentiality depends

The second most prevalent theme identified within our data, ‘Essentiality depends,’ was present in 43% of responses within the full sample and captured the sentiment that the essential nature of abortion as a medical procedure depends upon a pregnant person’s situation or the reason for seeking an abortion. Within this theme, responses most frequently indicated that abortion should be considered essential only when it is *medically necessary* (applied to 22.9% of responses within full sample). Most commonly, this included references to the health of the mother in determining essentiality: “I would think the only way it could be considered essential is if the mother would die otherwise,” while health of the fetus was mentioned less frequently, usually in tandem with mother’s health, “if the baby or mothers life is in danger than it is essential.”

Some responses within this theme positioned abortion as not an essential medical procedure, but potentially medically necessary in certain situations, suggesting there may be nuance in how some conceptualize essentiality in relation to medical necessity; for example, “#1, the taking of a life is not an essential service of any kind. # 2. If the mother is in danger, then it becomes a choice for the mother. That might make it an essential medical service.” Additionally, others rationalized that abortions, for reasons other than medical necessity, are not essential medical procedures, narrowly defining medical necessity in terms of health risk, “I think that it is an essential medical service when there is a physical/mental health risk to the mother or child. Simply wanting it for any reason does not constitute an essential medical service.” Responses also described abortion as being an essential procedure in instances where the pregnancy results from *rape or incest* (present within 4.3% of responses within full sample): “abortion should not take place unless raped and then before or up to 6 weeks,” and “The only time abortion should be done is if there was rape or incest.”

Finally, some responses simply stated that the essentiality of abortion *depends on the reason* (present within 15.8% of responses within full sample): “Because it does not apply to every woman, It is a case by case basis,” “Because it would depend on the reason,” and “Depends on the situation. Each situation is different.” Other responses emphasized needing to know the specific circumstances surrounding an abortion decision to make a determination, “The correct term would be ‘sometimes’, not unsure. There isn’t enough information to make a valid decision.”

### Abortion is not essential

The third theme ‘Abortion is not essential’ (present in 37.2% of responses within full sample) captured reasoning for why abortion should not be considered an essential medical procedure. Reasons against essentiality were more varied than reasons for, as reflected within the higher number of individual codes comprising this theme by comparison. Thus, responses within this theme encompass a variety of rationales for why abortion should not be considered an essential medical procedure.

Many responses within this theme expressed a similar rationale that when pregnancy creates medical issues, abortion is still considered to be elective, and thus not an essential medical procedure. Thus, some nonessential responses alluded to medical procedures, but distinguished abortion from other medical procedures, such as describing abortion as exclusively elective, not medically necessary, not as important as other medical procedures, and something that could be delayed. Responses shared general sentiment that abortion is simply *not essential or medically necessary* (applied to 7.3% of responses within the full sample): “99% of the time it is not medically necessary,” “Because it just is NOT,” “It is not a medical emergency.” Responses also referred to abortion as an *elective procedure*, and thus nonessential (applied to 5.3% of responses within full sample): “Abortion is a selective procedure not an emergency procedure,” “Abortion is something you chose to have done and it’s not an emergency situation,” and “The vast majority of the time neither the baby nor mother’s life is in danger. It is optional all most always.” In contrast to those in the sample who reasoned that abortion is an essential procedure because it is time sensitive, a small proportion (1.5%) of responses within the full sample stated that abortion is not essential because *“it can wait*,*”* for example: “it does not need to be done immediately and there is a fair window of time for when you can get it done.” Related, responses stated that *other medical concerns are more important* than abortion (1.2% of responses within full sample), “There is really no reason for a woman to have an abortion. There are people with actual medical problems that need attention in comparison.”

An additional rationale provided among responses within this theme is that abortion is a moral issue, not a medical issue. In line with this rationale, abortion was described as a nonessential medical procedure and not a medical issue/healthcare because it is exclusively a morality or ethical issue. Indeed, some responses (applied to 8.5% of responses within full sample) included reasoning that *abortion is murder*: “Abortion is Murder plain and simple…it is Not an essential medical service,” “Because it ends a life,” “Essential medical service [is] to save a life, not end a life,” and “When is it ever right to murder a person? A baby is a baby inside or outside of the womb. It is not a ‘medical’ procedure, it is the ending of a life on purpose for the convenience of the woman. If a woman’s life is endanger [sic] b/c of child birth, they would do an emergency c-section which is faster than an abortion.” Similarly, though less frequently, some responses included arguments that abortion is not an essential procedure because the act of abortion is *morally wrong* (applied to 5.7% of responses within full sample): “Because I believe it is immoral,” “I think abortion is wrong therefore it’s not medically necessary.”

Responses framing abortion as a moral issue rather than a medical issue also discussed that abortion is not an essential medical procedure when it is sought as a means to “*avoid responsibility*” (applied to 5.8% of responses within full sample), reasoning that abortion is a result of moral failing or lack of decision-making on the part of the pregnant person: “Because abortion is a result (in most cases) of poor choices,” and “Because most people choose abortion simply because they don’t want the baby.” This also included responses that stated abortion should not be used as birth control, “If it is being used as birth control then it isn’t essential.”

Finally, some responses also mentioned *alternatives to abortion*, such as adoption (1.9% of responses within full sample), “I do not believe in abortions! There are plenty of people who cannot conceive that would love to adopt a baby.” These responses implicitly suggest a moral or ethical connation to abortion, potentially distancing it from a conceptualization of healthcare.

### Don’t know, unsure, or unable to decide

Our final theme, applied to only 3.7% of responses within the full sample, included responses in which participants stated that that they simply could not make a decision about abortion as an essential medical procedure, either because they did not know, were uncertain, or felt ill-equipped to make such a decision. For instance, some responses indicated participants did not know enough contextual information: “Too many variables involved,” “If the mothers life is at risk it may not be a situation where waiting can be considered. If baby is at risk can they wait. If it was someone in my family…?? just UNSURE.” Others could not make a decision because of lack of personal knowledge: “I do not [have] much knowledge about medical services,” and “Because I am not a doctor to determine that.” These responses suggest that some people require more information to make a determination on abortion as an essential procedure. Interestingly, while 22.5% of closed-ended responses indicated “unsure,” only a small number of open-ended responses within the full sample reflected some aspect of being “unsure” in the sense that participants could not make a distinction of essentiality. Instead, it seems that the “unsure” close-ended option primarily captured attitudes that abortion essentiality depends on certain circumstances or reasons for seeking abortion, and only a small proportion of people were truly unsure, did not know, or could not decide.

### Inconsistencies between closed-ended and open-ended responses

**Fig. 2:**
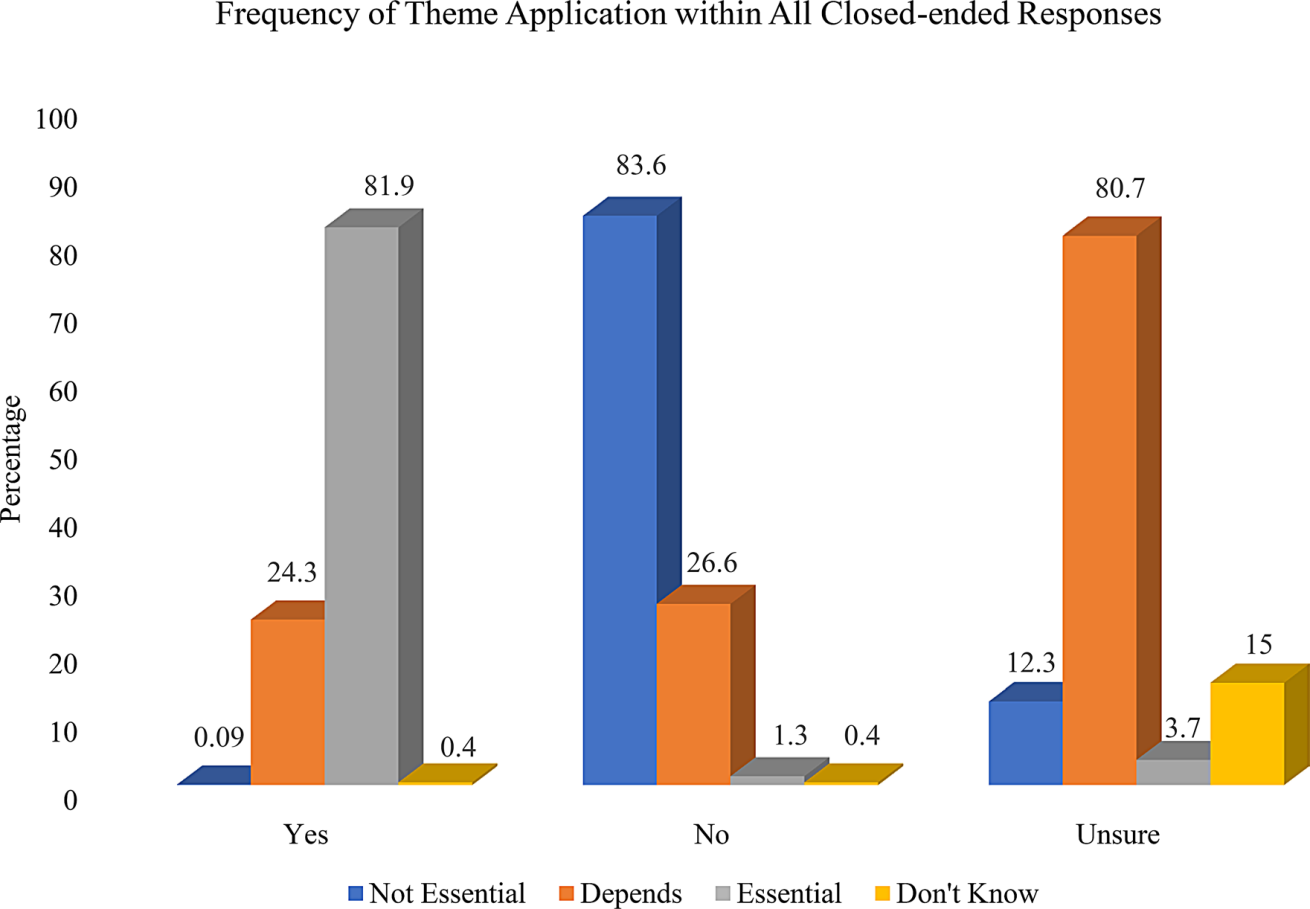
Frequency of theme application (Not essential, Depends, Essential, Don’t know/unsure/unable to decide) within Closed-ended Response Categories (Yes, No, Unsure)

During initial coding of open-ended responses, we noted several instances in which open-ended discussion of abortion as an essential medical procedure conflicted with closed-ended responses. For example, one participant who marked “no” to indicate that they did not think abortion was an essential medical procedure stated in their open-ended response, “I guess it really depends of [sic] the reason the abortion is being sought.” Thus, we also examined potential conflicts between closed-ended responses and open-ended responses. In Fig. 2, we report frequencies of theme application within each closed-ended response grouping, thus identifying the frequency of inconsistent responding between closed-ended response options. Because we allowed for application of multiple codes for each response, percentages total greater than 100.

Most open-ended responses corresponded to their closed-ended selection (e.g., the participant marked “no” to the closed-ended item and their open-ended response was coded from the theme ‘Abortion is not essential’). However, as can be seen in Fig. 2, some cases had codes applied to their open-ended responses that did not correspond to their closed-ended response. Most of these unaligned codes were in the “depends” category. For example, 24.3% of closed-ended responses which indicated “yes” abortion is an essential procedure, had a code from the ‘Essentially depends’ theme applied to the corresponding open-ended response. Similarly, 26.6% of the “no” closed-ended responses also had a code from the ‘Essentiality depends’ theme applied to the corresponding open-ended response. Additionally, approximately 10% of coded responses had codes applied from more than one theme, for example the response: “#1, the taking of a life is not an essential service of any kind. # 2. If the mother is in danger, then it becomes a choice for the mother. That might make it an essential medical service” simultaneously states that abortion is not essential (applied theme: ‘Abortion is not essential’) yet provides a reason for why the essentiality of abortion depends on a given situation (applied theme: ‘Essentiality Depends’). These findings highlight the complexity of abortion attitudes and the nuance required to capture attitudes with survey items.

The findings presented above include responses coded as ‘Essentiality depends’ and another coding theme, as well as responses only coded with an ‘Essentiality depends’ code. In Table 4, we reduced the sample to responses that were only coded as ‘Essentiality depends’ (i.e., did not have any secondary codes applied from the Not essential, Essential, or Don’t know/unsure/unable to decide themes). As expected, the largest proportion of responses coded as ‘Essentiality depends’ had a corresponding closed-ended response of unsure. However, meaningful proportions of responses which indicated yes and no on the close-ended item also provided open-ended responses coded as ‘Essentiality depends.’ Of note, the largest proportion of close-ended responses indicating either yes or no were associated with open-ended responses that referenced abortion being *essential if medically necessary* as the rationale for whether abortion is an essential medical procedure (see Table 4).

**Table 4:** Frequency of code application among responses coded as ‘Essentiality depends’ (*N* = 420)

	Yes (*N* = 116) *n* (%)	No (*N* = 71) *n* (%)	Unsure (*N* = 233) *n* (%)
Essentiality depends on the reason	22 (18.97)	17 (23.94)	164 (70.39)
Essential in cases of rape/incest	12 (10.34)	6 (8.45)	19 (8.15)
Essential if medically necessary	96 (82.76)	54 (76.05)	85 (36.48)

*Note. Sum of code applications can be greater than *N* and percentages can total greater than 100 because more than one code could be assigned to each response

## Discussion

We explored whether and how U.S. adults situate abortion as an essential medical procedure by analyzing responses to a survey question about whether abortion should be considered an essential medical procedure and participants’ corresponding rationale for holding this belief. Because we assessed participants’ perceptions, it is important to recognize that some rationales provided may not be grounded in scientific evidence, and indeed some people may even hold inaccurate or misinformed beliefs. For example, one respondent inaccurately believed, “If a woman’s life is endanger [sic] b/c of child birth, they would do an emergency c-section which is faster than an abortion.” Our findings revealed that, in response to the closed-ended item, just under half of participants indicated abortion is an essential medical procedure, while approximately one in three indicated abortion to not be an essential medical procedure, and approximately one in five were unsure. In exploring follow-up open-ended responses, we observed substantial variability. Interestingly, we noted complexity within responses, such that open-ended responses simultaneously indicated reasons both for and against essentiality and that a modest, but meaningful proportion of open-ended responses conflicted with their closed-ended responses regarding abortion essentiality.

The plurality of responses indicated that many participants believed abortion was an essential medical procedure, providing a variety of reasons for abortion essentiality. In explaining these reasons, responses primarily emphasized urgency, explaining that abortion is a time-sensitive procedure. This rationale is in line with statements from medical organizations released during COVID-19 defining abortion as an essential procedure due to time-sensitivity [20]. Public opinion research has also observed similar responses when surveying participants on telemedicine and medication abortion during COVID-19, with participants explaining their support for telemedicine to access medication abortion being due to the time-sensitive nature of abortion [31]. The second most-cited reason for abortion being an essential medical procedure was because it is an aspect of women’s healthcare or reproductive healthcare, with approximately one in ten responses mentioning that abortion is essential because it is healthcare. ACOG states that abortion is an “essential component of comprehensive health care” [1, 20], and our findings suggest that a small proportion of the U.S. public may conceptualize abortion as healthcare. That only one in ten responses explicitly mentioned abortion as healthcare conflicts with the perspective of relevant medical organizations and experts that conceptualize abortion as essential healthcare [20]. It is important to note that we asked about abortion as an essential *medical* procedure and as such people may have implicitly assumed abortion is healthcare when providing their open-ended response. Indeed, because we did not directly ask about perceptions of abortion as healthcare, we cannot say for certain whether only this small proportion of the public conceptualize abortion in this way. It may be that people think about abortion in a variety of ways—perhaps as healthcare, but in other ways as well.

To that end, approximately one third of responses indicated that abortion should not be considered an essential medical procedure. Open-ended responses coded within the theme of ‘Abortion is not essential’ demonstrated a greater variety of reasons, underscoring the broad and varied ways people may conceptualize abortion. Most prominently, responses mentioned the moral nature of abortion—describing it as murder or taking a life and as immoral, negating essentiality, and by extension, the nature of abortion as healthcare. Such findings are consistent with polling data suggesting that a substantial proportion of people conceive abortion as immoral [25]. Conceptualizing abortion as immoral, rather than as an essential medical procedure, could contribute to abortion stigma experienced by those seeking as well as providing abortion which in turn presents harmful health outcomes, such as psychological distress, among abortion seekers [32, 33].

Disregarding morality, several responses indicated that abortion is not an essential medical procedure because it is an “elective” procedure, that it can be delayed, or that other medical issues take precedence over abortion. Such attitudes may reflect a lack of knowledge of state-level gestational limits on abortion or knowledge of fetal development in the context of gestational timing. For example, U.S. adults, particularly those in conservative states such as Mississippi and Texas, report being unaware of abortion policies in their state [34, 35]. Additionally, U.S. adults also show misunderstanding of when a person becomes aware they are pregnant [36]. Abortion knowledge notwithstanding, these responses seem to acknowledge abortion as being situated within a medical domain, but perhaps not a procedure that should be prioritized during a situation such as the COVID-19 pandemic. In that vein, perhaps such participants may conceptualize abortion as healthcare, but not “important enough” healthcare. Participants’ framing of abortion as an elective procedure also reflects narratives within newspaper articles published, particularly within the U.S. South, during the pandemic that framed abortion as an individual choice (and thus elective) and as a public health threat due to disruption of pandemic response efforts [37]. Such perspectives fail to consider structural barriers impacting abortion choice [37] and may perpetuate abortion-related stigma that presents barriers to reproductive healthcare [38] and contributes to negative health outcomes such as psychological distress and somatic symptoms [39].

Indeed, discussion of abortion as being elective, or not of great medical importance, is a key aspect to abortion discourse. For decades abortion has been framed across news media and scholarly literature in different ways, such as “elective” versus “indicated” or “traumatic” when comparing, for example, abortion sought for relational or financial reasons (i.e., sometimes described as elective) and abortion sought for reasons such as maternal health or fetal anomaly (i.e., sometimes described as indicated or traumatic) [40–42]. Some within the pro-life/anti-abortion movement also position some interventions that may lead to fetal demise as understandable if there are good effects and intentions as articulated through the moral principle of double effect. For instance, according to the National Catholic Bioethics Center, engaging in procedures that may lead to fetal termination can be understandable if they are implemented to save the life of the pregnant person [43]. Additionally, OBGYNs themselves, when interviewed, report distinguishing “indicated” and “elective” abortions, viewing “indicated” abortions (i.e., abortions sought when the fetus is unhealthy) as more legitimate abortions than elective abortions [44]. However, making consistent and transparent distinctions between “elective” versus “indicated” abortions is difficult to realistically carry out in practice in part due to the myriad reasons people seek abortion [45, 46]. Scholars have argued that referring to abortion as elective “imparts moral judgement and perpetuates abortion stigma” ([40] p 89) and may serve to constrain abortion access by aligning with legal and regulatory practices that aim to restrict abortion for elective reasons [44]. Keeping this in mind, it is important to note that the present data were collected during the COVID-19 pandemic for the purpose of understanding people’s attitudes about abortion in a situation when many medical procedures were being limited.

The small proportion of responses which indicated that abortion can be delayed is interesting in comparison with the much larger proportion that reasoned abortion is time sensitive and thus essential. As many responses noted, abortion is physiologically time sensitive because pregnancy is a temporary state. Additionally, participants noted that there is a legislative window which necessitates timing in terms of abortion. Similarly, people are far more inclined to endorse legal abortion earlier in pregnancy than later [47–49], adding an additional timing implication. Regarding ability to delay abortion procedures, the American Association for the Surgery of Trauma (AAST) identified clinical conditions that constitute the necessity of emergency general surgery, and unwanted pregnancy does not appear on this list. However, pregnancy can lead to a plethora of medical conditions for pregnant people, such as hernia, intestinal obstructions, and cardiovascular issues [50]. As such, practically, it can be difficult for healthcare providers to navigate the extent that abortion is an essential medical procedure in some instances due to these potential outcomes. This can extend to public sentiment given that many of our participants conditioned essentiality on the extent that pregnancy may result in negative health implications.

Importantly, our findings revealed complexity in reasons for thinking about abortion as essential versus nonessential. Within our theme ‘Essentiality depends,’ open-ended responses most prevalently indicated that abortion is only an essential medical procedure if it is “medically necessary,” such as in instances where the health or lives of the pregnant person or fetus are in danger by continuing a pregnancy. Responses also indicated that abortion may be considered an essential medical procedure in cases of pregnancy resulting from rape or incest. These attitudes mirror laws restricting or banning abortion than allow for exceptions in cases of rape-related pregnancy [51]. These views on the nature of abortion as an essential medical procedure are also in line with research on attitudes toward abortion legality in which people are more inclined to endorse legal abortion when abortion is sought for medical or rape-related reasons as opposed to reasons pertaining to not being able to afford or not wanting (more) children [48, 52, 53]. This framing of some reasons for abortion as more valid or acceptable than others creates a hierarchy of potential reasons for seeking abortion which was also reinforced by media outlets during COVID-19 [37].

Also in line with research regarding attitudes toward abortion legality [27], some responses indicated needing more information or additional context to make a determination of medical essentiality, basing this determination on reasons for seeking abortion or other conditions surrounding the pregnancy, pregnant person, and the abortion. Many responses mentioned that determining the essentiality of abortion as a medical procedure should be done on a case-by-case basis which was not reflected in blanket bans of abortion services, such as decisions made early in the pandemic (e.g., Ohio) and would likely be challenging to implement. Alternatively, a small number of responses indicated a lack of ability to make a determination regarding abortion as an essential medical procedure. These in-between and conflicting responses regarding abortion as an essential medical procedure are relevant when applying our findings to considerations of abortion as healthcare.

Interestingly, we noted instances of discrepancy between closed-ended and open-ended responses in conceptualizing abortion as an essential medical procedure. The belief that abortion is an aspect of reproductive healthcare emerged as rationale for abortion being essential, yet, in contrast, responses indicating abortion was not essential seemed to argue against abortion as healthcare, and those who said essentiality depends seemed to cite “medical necessities” to qualify abortion as healthcare. These discrete reasons tended to address maternal and/or fetal morbidity and morality, suggesting a somewhat narrow conceptualization of what may qualify as an essential medical procedure and thus potentially healthcare. In many instances, participants indicated abortion was not an essential medical procedure in closed-ended responses, yet their companion open-ended responses elaborated that abortion may be considered essential if it is “medically necessary.” This variation in responding may have been observed given that U.S. adults are generally more accepting of abortion occurring in the context of health endangerment and severe fetal anomalies [53], contexts in which abortion may be considered “medically necessary.” In addition, the ability to provide an open-ended response to the question of whether abortion is medically essential could have provided participants with the opportunity to share more nuanced or complex attitudes that were unable to be captured by the limited closed-ended response options. Thus, it would seem that some people believe abortion can be healthcare in limited contexts, such as when someone’s life is in danger.

These varied determinations of the essentiality of abortion as a medical procedure reflect the notion of abortion exceptionalism which posits that abortion is regulated more stringently than other medical procedures at comparable levels of complexity, which can be extended to understanding the stigmatization of abortion and conceptualization of abortion as healthcare in U.S. society, with such understandings being compounded by COVID-19 [54]. Other health issues are generally not subject to these biological, legal, and social restrictions, which separates abortion from other forms of healthcare and perhaps informs people’s complicated conceptualizations of abortion. Whether abortion is perceived as healthcare by U.S. adults can, for some, depend on the circumstances surrounding abortion. These complex attitudes toward abortion as an essential medical procedure are in line with prior research evidencing the nuance in attitudes toward abortion based on various contextual factors [27, 52, 55–58]. And importantly, collectively, these findings suggest a divide in conceptualizing abortion that may be relevant for advocates to consider, as messages pertaining to abortion as healthcare may simply not resonate with a certain proportion of the population.

### Limitations & future directions

The present study should be considered in light of several limitations. First, frequencies of open-ended responses may not be representative to the U.S. population, but because we used probability-based sampling, the sentiment of the responses is likely generally representative. Nevertheless, generalizability of our open-ended findings to the U.S. public as a whole is limited. Second, although we provided the following context, “*During the coronavirus outbreak*,* medical appointments were limited to****only those considered essential***,*”* there may have been the potential for between-person variation in understanding or conceptualization of the term “essential” among participants. In addition, given that our questions framed abortion as a medical procedure, participants’ conceptualizations of abortion in general, as healthcare more broadly, or as care tangential to healthcare, such as community care, may have been limited. Furthermore, data were collected from September 2020 to January 2021, months after the COVID-19 pandemic initial outbreak in the U.S. and thus months after immediate changes to healthcare legislature were occurring broadly. As such, specific policy changes related to abortion may not have been as salient to participants. Additionally, as discussed prior, determinations of abortion essentiality were made on a state-by-state basis; however, we did not collate the data by state to observe any potential differences in attitudes toward abortion as essential based on state. Given the specificity of the present findings to the COVID-19 pandemic context, it would also be important to assess adults’ attitudes again after the pandemic as some of the responses related to abortion being elective, or not as important as other medical procedures, could have been in reference to the conditions of the pandemic, limiting the extent they reflect people’s perceptions of abortion as healthcare. In doing so, given the proportion of people whose open-ended response indicated that their perception of abortion as an essential medical procedure “depends,” it may be fruitful for researchers to consider providing alternative response options to our closed-ended question such as “sometimes,” or “it depends on the circumstance,” or even provide different contexts consistent with the GSS.

## Conclusions

Although the plurality of analyzed responses indicated abortion to be an essential medical procedure during COVID-19, meaningful proportions of responses also reported abortion to be nonessential for moral reasons or because they perceived abortion to be a less important, elective procedure. There was also a significant proportion of responses which explained that there are only certain situations in which abortion should be considered an essential medical procedure, particularly when health is endangered. We noted complexity in conceptualizations of abortion as an essential medical procedure, and thus an aspect of healthcare, across all three close-ended response groups (i.e., those who said abortion is essential, is not essential, or were unsure), with some beliefs and reasoning conflicting. Indeed, at least some people across all three groups provided rationales suggesting they did not think abortion (or all abortions) were essential or “essential enough,” potentially distinguishing abortion from healthcare. And among those who said essentiality depends specifically in their close-ended response, many seemed to limit their conceptualization of abortion as an aspect of healthcare based on the reasons for seeking abortion (e.g., “health reasons”), thus distinguishing notions of essentiality and potentially healthcare based on context. Thus, it is possible that blanket statements like “abortion is healthcare” may not resonate with people holding these perspectives. Additional research specifically examining people’s conceptualization of abortion as healthcare and the impact such slogans have on people’s attitudes or attitude formation may be fruitful. It is important to note that the proportion of adults perceiving abortion as an essential medical procedure may have been reduced by the conditions of the pandemic when other important procedures (e.g., some peripheral vascular disease and cancer surgeries) were also classified as elective by medical professionals. Abortion as an essential medical procedure may have increased as the pandemic dissipated, and thus, continued research examining people’s understanding and appreciation of “abortion as healthcare” would be valuable.

## Data Availability

The data that support the findings of this study may be available from the corresponding author [KNJ], upon reasonable request.

## Footnotes

1 Across participants who completed at least one survey across Waves 2–4, 68 participants did not provide an open-ended response at any wave (i.e., either did not complete the survey at a given wave or failed to provide an open-ended response at a given wave or all of the waves), and thus were excluded from analysis. Demographic characteristics for these 68 participants did not vary from that of the included 643 participants.

2 Data was weighted to match the US benchmarks of gender, age, race/ethnicity, education, census region, household income, home ownership, metropolitan areas, and Hispanic origin.

3 According to Hawbaker, et al., (2023), medicality is defined as “having an attribute or condition related to medicine – or as being related to the formalized system of Western medicine” ([30] p 1).
